# Gene expression profiling of postnatal lung development in the marsupial gray short-tailed opossum (*Monodelphis domestica*) highlights conserved developmental pathways and specific characteristics during lung organogenesis

**DOI:** 10.1186/s12864-018-5102-2

**Published:** 2018-10-05

**Authors:** Vengamanaidu Modepalli, Amit Kumar, Julie A Sharp, Norman R Saunders, Kevin R Nicholas, Christophe Lefèvre

**Affiliations:** 1 School of Medicine, Deakin University, Pigdons Road, Geelong, VIC Australia; 2 Department of Anatomy and Developmental Biology, Monash University, Clayton, Australia; 3 Division of Bioinformatics, Walter and Eliza Hall Medical Research Institute, Melbourne, Australia; 4 Department of Pharmacology and Therapeutics, The University of Melbourne, Melbourne, Australia; 5 Peter MacCallum Cancer Centre, Melbourne, Australia; 6 Institute of Frontiers Materials, Deakin University, Pigdons Road, Geelong, VIC Australia; 7 Monash Institute of Pharmaceutical Science, Faculty of Pharmacy and Pharmaceutical Sciences, Monash University, Parkville, VIC 3052 Australia

**Keywords:** Lung, RNA-seq, *Monodelphis domestica*, Marsupial

## Abstract

**Background:**

After a short gestation, marsupials give birth to immature neonates with lungs that are not fully developed and in early life the neonate partially relies on gas exchange through the skin. Therefore, significant lung development occurs after birth in marsupials in contrast to eutherian mammals such as humans and mice where lung development occurs predominantly in the embryo. To explore the mechanisms of marsupial lung development in comparison to eutherians, morphological and gene expression analysis were conducted in the gray short-tailed opossum (*Monodelphis domestica)*.

**Results:**

Postnatal lung development of *Monodelphis* involves three key stages of development: (i) transition from late canalicular to early saccular stages, (ii) saccular and (iii) alveolar stages, similar to developmental stages overlapping the embryonic and perinatal period in eutherians. Differentially expressed genes were identified and correlated with developmental stages. Functional categories included growth factors, extracellular matrix protein (ECMs), transcriptional factors and signalling pathways related to branching morphogenesis, alveologenesis and vascularisation. Comparison with published data on mice highlighted the conserved importance of extracellular matrix remodelling and signalling pathways such as Wnt, Notch, IGF, TGFβ, retinoic acid and angiopoietin. The comparison also revealed changes in the mammalian gene expression program associated with the initiation of alveologenesis and birth, pointing to subtle differences between the non-functional embryonic lung of the eutherian mouse and the partially functional developing lung of the marsupial *Monodelphis* neonates. The data also highlighted a subset of contractile proteins specifically expressed in *Monodelphis* during and after alveologenesis.

**Conclusion:**

The results provide insights into marsupial lung development and support the potential of the marsupial model of postnatal development towards better understanding of the evolution of the mammalian bronchioalveolar lung.

**Electronic supplementary material:**

The online version of this article (10.1186/s12864-018-5102-2) contains supplementary material, which is available to authorized users.

## Background

During evolution mammals have developed a bronchoalveolar lung characterised in part by the presence of a large number of small alveoli. Eutherians and marsupials have evolved from common therian mammalian ancestors, but adopted different reproduction strategies [1]. Eutherians, including humans and mice, acquired a well-developed placenta which sustains the embryo throughout a long period of gestation; they give birth to a mature neonate [2]. In contrast, marsupials have retained a primitive form of placenta and after a short gestation give birth to immature neonates [3]. Therefore, organs in the marsupial newborns are generally at comparatively earlier stages of development at birth and the respiratory, digestive, neuronal, immune and respiratory systems are immature and still under the process of development [4].

In eutherians the lungs develop as a respiratory organ to exchange gases immediately after birth. In general, the majority of lung development occurs throughout intrauterine life and is driven, in part, by maternal factors delivered through the placenta [5–7]. Lung development is categorized into five morphological stages (embryonic, pseudoglandular, canalicular, saccular and alveolar) based on characteristic morphology [8, 9]. In eutherians the lungs of the newborn are predominantly at the transition between the saccular and alveolar stage of development with small terminal sacs and a well-developed bronchial system and the key changes during early postnatal life involve an increase in alveolar number and maturation of lung microvasculature [10]. Studies on the development of the lung have been performed previously in several marsupial species, including bandicoot (*Isoodon macrounus*) [11], Julia Creek dunnart (*Sminthopsis douglasi*) [12], tammar wallaby (*Macropus eugenii*), quokka (*Setonix brachyurus*) [13] and *Monodelphis domestica* [14]. At birth, the lungs are comprised of a small number of large air sacs providing limited surface area for respiration and are therefore considered functionally immature [15]. Studies of the respiratory mechanism of Julia Creek dunnart and tammar wallaby have demonstrated that neonates perform respiration through the skin during early postnatal development in order to fulfil the requirement for oxygen. However, this limited development changes gradually as lungs mature to perform efficient respiration [12, 16]. Marsupial neonates are similar in development to a late eutherian foetus corresponding to the 40–100 day old human foetal stage, foetal rat at E13-E14 or mice E12-E13 [17, 18], and the immature lung is required to develop further during early lactation to become fully functional. This provides improved opportunities to investigate the progressive changes in the gene expression of the postnatal lung and identify mechanisms and factors involved in lung maturation.

## Results

### Postnatal lung development in *Monodelphis*

**Fig. 1:**
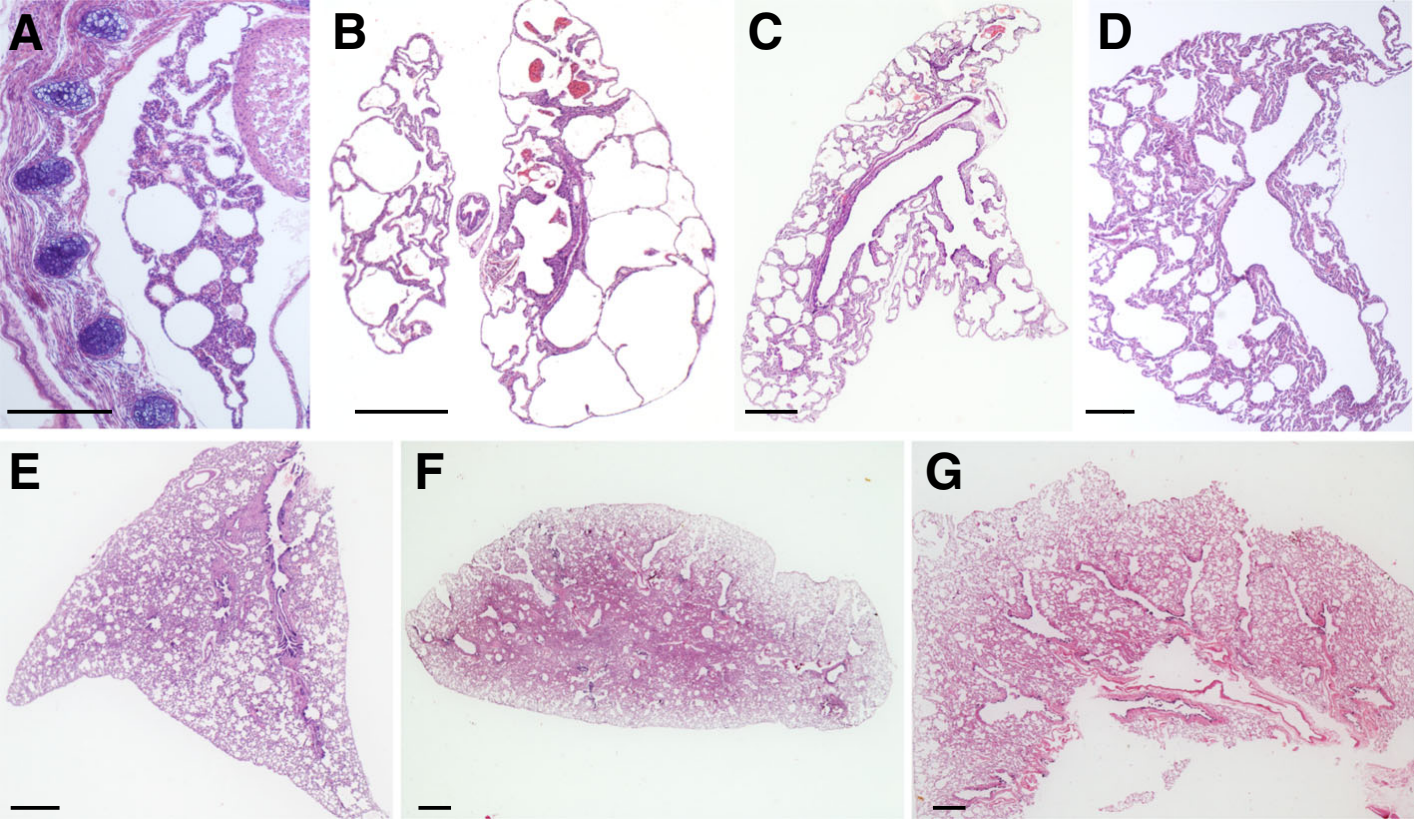
H&E staining of lung tissue samples collected from *Monodelphis* at different time points of postnatal development. Based on morphology as described in the text, the lungs of new born at day 1 (**a**) were in the canalicular stage, lungs collected at day 8 (**b**) and day 14 (**c**) were at early saccular stage, and at saccular stage by day 29 (**d**) and day 35 (**e**), while at day 61 (**f**) lungs were mature with an increased alveolar number similar to an adult lung (**g**). **a** Is a full embryo cross-section. Whole lung mounts (**b** to **f**), scale bar 1 mm. See also Additional file 1: Figure S1 for pictures at higher resolution where individual cell nuclei are easier to identify

Histology of lung samples collected from *Monodelphis* at post-natal days 1, 8, 14, 29, 35, 61 and adult stages (Fig. 1 and Additional file 1: Figure S1 for high resolution images) revealed that the lungs collected 1 day after birth consisted only of a few large air sacs surrounded by a thick layer of epithelial cells (Fig. 1a) typical of the late canalicular stage of development. Lung collected at day 8 consisted of large air sacs and the thickness of the walls had reduced (Fig. 1b). The sacs were lined with a single layer of cells and were connected with a single respiratory duct, indicating the lungs were transiting from the canalicular to the saccular stage. The number of air sacs had increased in the lungs collected at day 14 and the sacs were comparatively smaller in size, but the thickness of the air sac walls had increased and only partial regions consisted of a thin layer of epithelial cells (Fig. 1c). At day 29 the number of air sacs had increased and the airways extended into the terminal air sacs. The increase in the respiratory area by growth of air sacs was correlated with a gradual decrease of interstitial tissue (Fig. 1d). At day 35 the lungs were transitioning from the saccular to the alveolar stage with primitive alveolar sacs connected to air ducts (Fig. 1e). At this stage, the airway system and branching pattern was established throughout the lung. Finally, after a large increase in the number of alveoli at day 63 the lung was fully mature (Fig. 1f) and histological analysis showed no major difference in morphology in lungs from the adult (Fig. 1g). The results are consistent with previous studies, confirming that lung development follows similar trajectory in all mammals examined and providing imaging at additional time points to improve our understanding of the slower developmental timing in *Monodelphis* and other marsupials compared to eutherians [14].

### Transcriptomics of postnatal lung development in *Monodelphis*

**Fig. 2:**
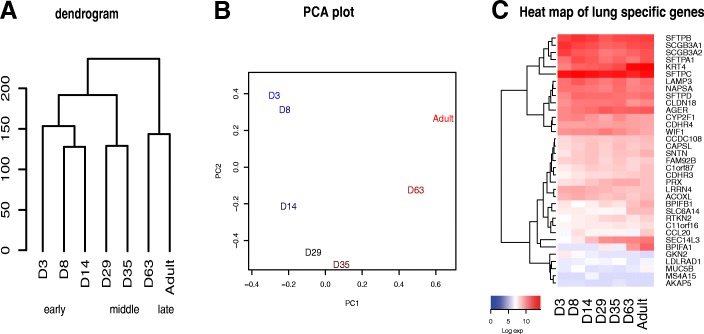
Global gene expression analysis of *Monodelphis* lung. **a** Hierarchical clustering dendogram of postnatal log expression data. **b** First two principal components PCA plot. **c** Heatmap of lung specific gene expression

Gene expression profiling of *Monodelphis* lung was conducted by RNA-seq of lung tissue collected from young at day 3, 8, 14, 29, 35, 61 and adults. After annotation and normalisation as described in methods, hierarchical clustering of the normalised quantitative expression data (Fig. 2a) suggested 3 major groups of samples, corresponding to three phases of lung development: 1) early postnatal development (day 3, 8 and 14), 2) mid-development (day 29 and 35), and 3) late development (day 63 and adult). This result was consistent with morphological analysis and confirmed that there are two major transitions in gene expression corresponding to the early canalicular-to-saccular and saccular-to-alveolar morphology. Principal component analysis (PCA) indicated that the greatest influence on gene expression was the age of the animal (51% of variation) while the second greatest component (21% of variation) was apparently influenced by time to or from the transition into alveogenesis (Fig. 2b).

### Surfactant proteins and highly expressed genes relevant to lung function

The surfactant protein genes *SFTPB* and *SFTPC* were amongst the most highly expressed genes in the RNA-seq dataset overall (rank max log expression 62 and 29 in any sample respectively; rank mean log expression 84 and 22) with a relatively constant expression throughout development. RNA-seq also confirmed high expression of surfactant *SFTPA* and *SFTPD* candidate genes on chromosome 1 throughout the postnatal period (Fig. 2c). Similarly, osteonectin (*SPARC*) encoding an acidic extracellular matrix glycoprotein that plays a vital role in cell-matrix interactions and collagen binding, was among the 26 most highly expressed genes. The top 100 expressed genes overall were, in majority, mitochondrial and ribosomal proteins genes but included keratins (15, 19, 35 and 4) with increasing expression during development (for example *KRT4* in Figs. 2c and 5b), and NADPH oxidase organizer 1 (*NOXO1)*, a regulator of angiogenic capacity of lung endothelial cells [19] with constant expression. Other genes of relevance to lung physiology that were highly expressed (top 200 genes) included secretoglobin family 3A member 1 and 2 (*SCGB3A1, SCGB3A2,* Fig. 2c), resistin like beta (*RETNLB*), a mitogenic factor in lung cell induced in hypoxia, decorin (*DCN*), an extracellular matrix proteoglycan which affects airway mechanics, airway-parenchymal interdependence, airway smooth muscle proliferation, apoptosis and transforming growth factor-β bioavailability, *ADA* (adenosine deaminase), which has been associated with pulmonary inflammation [20], and *EPAS1* (endothelial PAS domain protein 1) which is potentially involved in lung and vascular development [21].

### Lung-specific gene expression

A recent comparative study of gene expression in different eutherian tissues has identified a set of 83 candidate genes with lung-specific expression, including 32 confirmed genes [22]. In *Monodelphis,* 20 out of the 32 confirmed gene set and 35 out of the 83 candidate gene set were identified and presented a variety of expression dynamics (heatmap of Fig. 2c). It is not clear if the remaining lung-specific candidate genes are not expressed in the lung, not yet annotated or absent from the *Monodelphis* genome.

### Differential expression

**Fig. 3:**
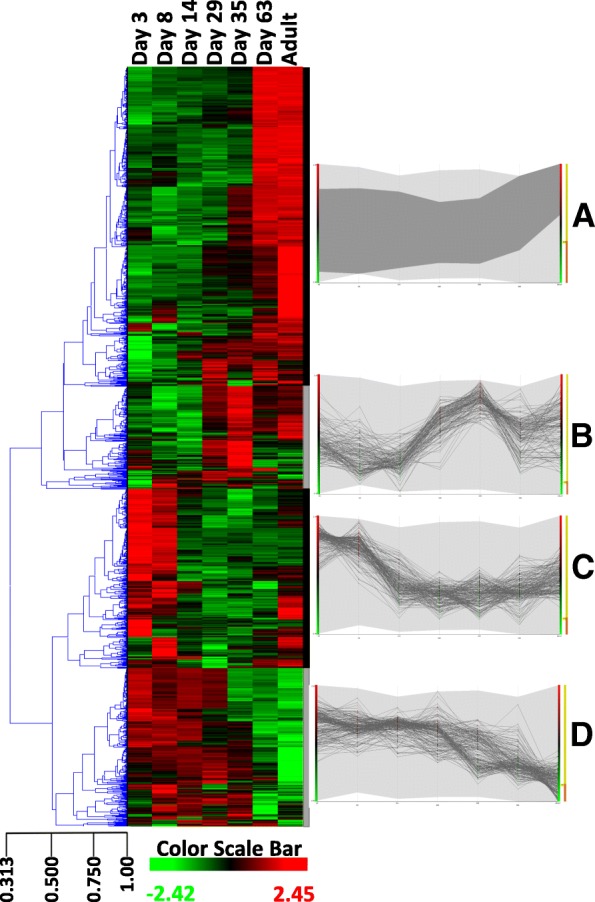
Heatmap and hierarchical clustering of 1242 genes with the most significantly differential expression during postnatal lung development (*P* < 0.05). Centred and scaled log RNA-seq expression data. Rows in the heatmap represent genes and columns represent samples. The gene tree on the left was generated by hierarchical clustering. Four gene clusters were selected to represent genes with different expression dynamics (k-mean clustering, k = 4, right side). Expression profile of the four major gene expression clusters was during (**a**) the later alveolar stage of development, **b** saccular stage of development, **c** earlier postnatal period with a gradual reduction of expression during the course of development, and (**d**) early stages of development. The limits of each cluster are represented by grey and white bars along the right side of the heatmap. Cluster A is the largest gene cluster and the expression profile of this cluster is summarized by a dark grey band (min, max log expression) rather than showing independent gene expression as in cluster (**b** to **d)**. The light grey shaded area represents the range log expression of all the data (1242 genes)

**Fig. 4:**
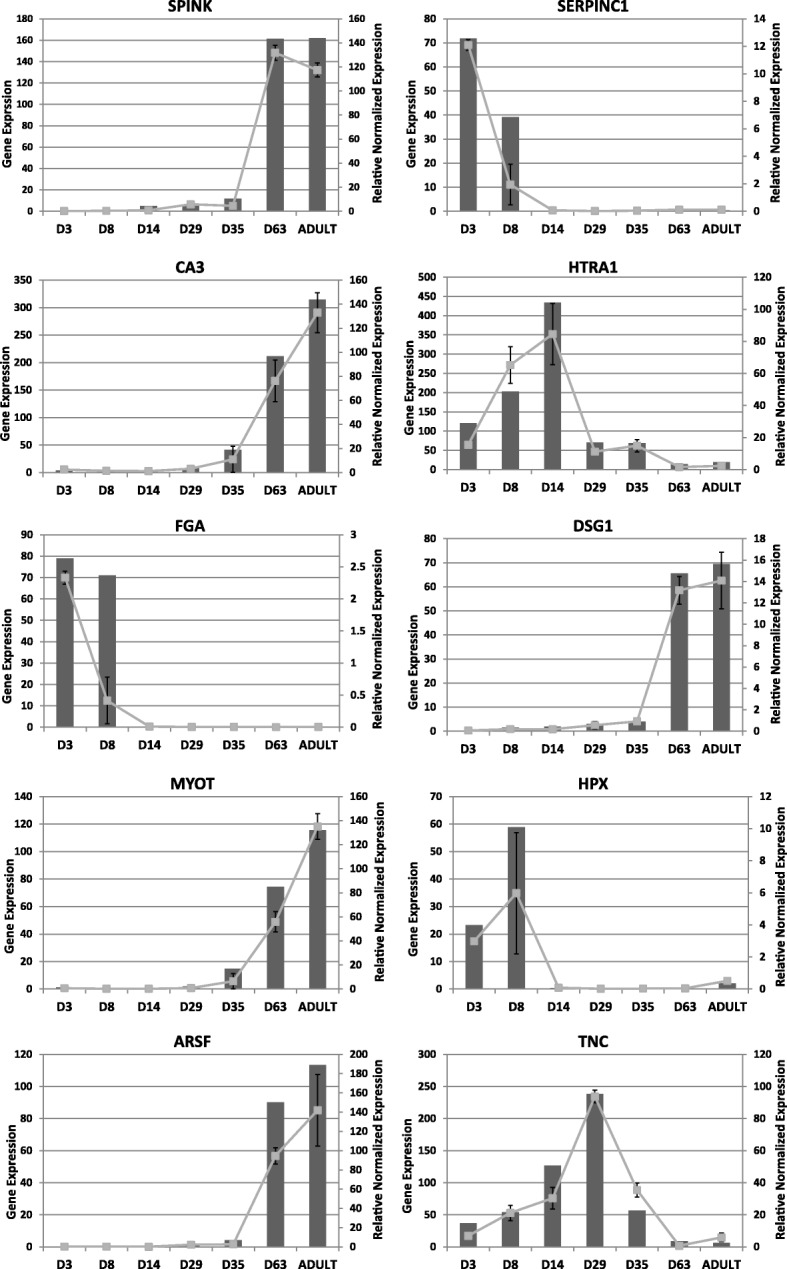
Validation of RNA-seq data. Comparison of gene expression profiles obtained by q-PCR (line) and RNA-seq quantification (bar). For this validation, 10 genes were chosen to represent expression dynamics of clusters identified in Fig. 3. This includes genes from cluster A (SPINK5, CA3, ARSF, MYOTand DSG1), cluster C (HPX, FGA, SERPINC1) and cluster D (TNC, HTRA1) in Fig. 3. In general, there is high concordance between expression results from q-PCR and RNA-seq data

**Fig. 5:**
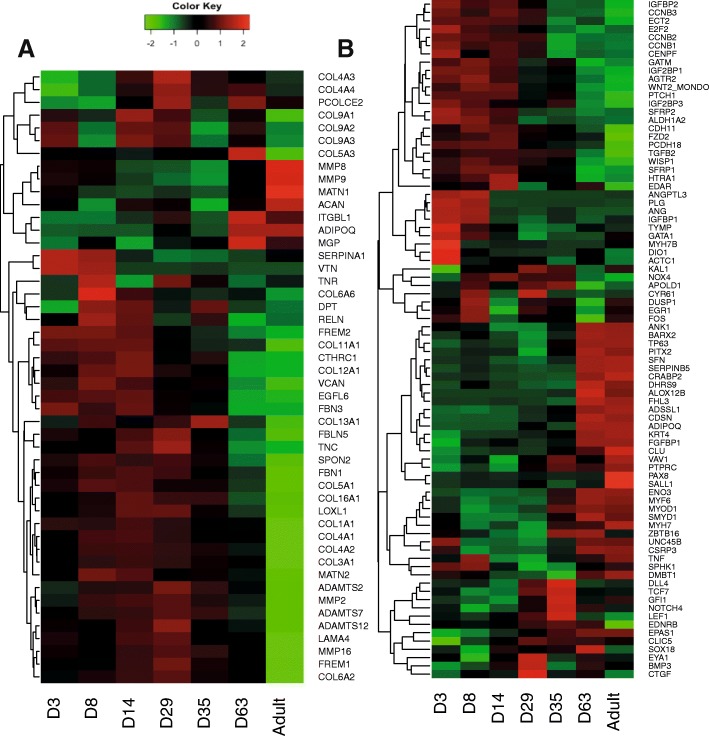
Differentially expressed genes related to lung morphology and development. Heat map (centered and scaled RNA-seq expression data) showing the expression profile of genes related to (**a**) the extracellular matrix (ECM and MMPs) and (**b**) regulatory factors (signalling molecules and transcriptional factors). Rows represent genes and columns represent development phases. Gene symbols are listed on the right side. While a majority of ECM molecules exhibit higher expression during development (day 3 to 35), regulatory molecules are either highly expressed during early (top), intermediate (bottom) or late (middle) phases of development

Differential expression analysis revealed 1242 genes were uniquely or differentially expressed at specific time points during postnatal development as represented in the heat map of Fig. 3 (*p* < 0.05). Sequencing results were validated by RT-PCR as described in methods. A set of 10 genes was selected to represent differential expression clusters (genes chosen from profiling of Fig. 3). Although this is a limited validation of 10 genes and there are a few discrepancies at particular time points such as, for example, day 8 for SERPINC1 (serpin family C member 1), FGA (fibrinogen alpha chain) and HPX (hemopexin), in general there is a high concordance between the temporal expression trends obtained by qPCR and RNA-seq results (Fig. 4). It is unclear if discrepancies are due to variation between animals with a single animal used for sequencing or transitional variation around particular time points of rapid changes such as day 8 for SERPINC1 and FGA. Functional classification of differentially expressed genes highlighted genes encoding for components of cellular processes (446 genes), binding activity (363 genes), developmental processes (298 genes) and immunity (273 genes) (Additional file 2: Figure S2). Developmental processes included system development (190 genes) related to various systems (nervous system, heart, muscle and haematopoiesis). Associated functional categories were also related to embryonic development (ectoderm and mesoderm) and cell differentiation. Cellular processes were mainly related to cell communication (251 genes) while others were related to cell cycle and proliferation. Many (44%) of the genes listed in response to stimulus were related to immune (114 genes) and cellular defence responses (57 genes). Genes involved in signalling included components of the WNT, PDGF, TGFβ, NOTCH, VEGF and Retinoic acid (RA) pathways (see discussion in Additional file 3). Differential expression of genes related to the extracellular matrix (ECM) and signalling (also discussed in Additional file 3) are represented in Fig. 5a and b respectively. It can be seen that these genes exhibit a variety of expression profiles and that a broader subset of ECM components display decreased expression over time, likely to reflect the active establishment of complex tissue connectivity in early stages.

### Gene expression clustering

Gene expression clustering of differentially expressed genes produced 4 major temporal expression patterns (Fig. 3, clusters A, B, C and D). A large set of genes presented a gradual increase in expression as the lung developed (cluster A, 523 genes) with high expression during the later alveolar stage at day 63 and in the adult. This gene set was enriched in genes associated with muscle contraction (enrichment score (ES) 8, *p* = 2E-16), calcium binding (ES 4, *p* = 1.2E-5) and epithelial development (ES 4, *p* = 1.4E-4). In contrast, a second set of genes was specifically expressed during the early phases of development with lower expression as lung matured (cluster C, 293 genes). This cluster was enriched in secreted and extracellular matrix components (ES 18, *p* = 1.5E-28), plasma proteins, innate immunity and response to wound healing (ES 6, *p* = E-31 ~ E-13). Other genes were specifically expressed at particular time points, mainly including day 14, 29 and 35, with increasing (cluster B) or decreasing (cluster D) expression during the course of development. Cluster B (166 genes with peak expression at saccular stage days 29 and 35, Fig. 3b) was enriched in immune genes involved in T cell activation (ES 7, *p* = 1E-17), cell surface signalling (ES 4, *p* = 4.5E-7) and the regulation of apoptosis (ES 3, *p* = 8.4E-5). Finally, cluster D contained 260 genes highly expressed during early stages from day 3 to day 29 with decreased expression from day 35 (Fig. 3d) and was enriched in extracellular matrix (ES 17, *p* = 6E-25) and cell cycle (ES 8, *p* = 1.7E-12). Overall the results confirm that temporal differential gene expression is associated with morphological changes during development, revealing gene markers and providing insight into the development processes with active cell division, extracellular matrix deposition and establishment of innate immunity in early development followed by establishment of adaptive immunity and, finally, epithelial proliferation and, more surprisingly, muscle development.

### Comparative analysis of lung development transcriptomes in *Monodelphis* and mice

Published gene expression studies of lung development in mouse [23] and human [24] lung tissue have previously identified a molecular signature of time-to-birth supported by principal component analysis. In *Monodelphis*, principal component analysis similarly indicated that the first component represented the age of the animal (Fig. 2b). However, in this case the second component was not influenced by time from birth but was instead apparently influenced by the time to and from the saccular-alveolar transition, a period overlapping the time of birth in mice but occurring only after birth around day 35 postnatal in *Monodelphis*.

**Fig. 6:**
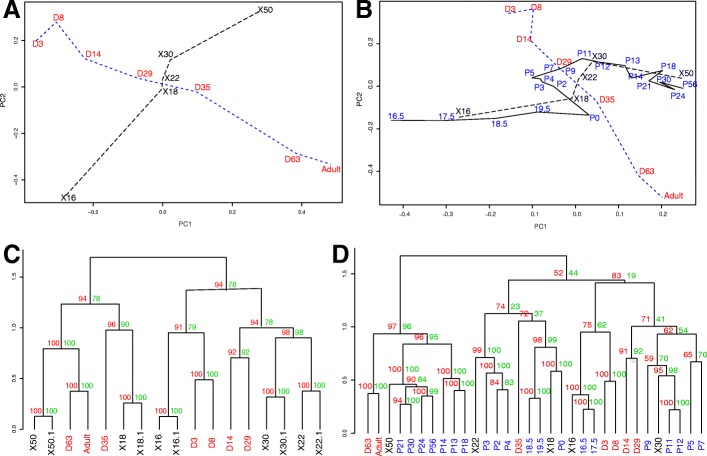
**a** Principal Component Analysis and clustering of the combined gene expression datasets from *Monodelphis* (red, postnatal day D3, D8, D14, D29, D35, D63 and adult) and mice GSE20954 (black, days post-conception X18, X18, X22, X30 and X50). **b** Principal Component Analysis of the combined gene expression datasets in A plus a second, more comprehensive, mice dataset GSE74243 (blue: embryonic day 16.5 to 19.5, postnatal day P0 to P56, average expression of replicates at each time point for the B6 strain). **c**, **d** Hierarchical co-clustering and bootstrap values of combined data in A and B (correlation distance, clustering method complete). The tree is annotated with Bootstrap Probability (green) and Approximately Unbiased *p*-value (red)

To compare further the mammalian lung development programs of *Monodelphis* and mice, two murine gene expression microarray datasets were retrieved from the Gene Expression Omnibus database (GEO dataset GSE20954 and GSE74243) [25, 26]. Combining *Monodelphis* RNA-seq and mice microarray dataset GSE20954, the first principal component (37% variation) remained mainly influenced by age, providing an approximate alignment of development time between the species (Fig. 6a). Not surprisingly, the second component (22% variation) represented variation between species. Clustering of the combined dataset resulted in co-clustering of the two species at: 1) early time points (mouse day E16 and *Monodelphis* days 3 and 8), 2) intermediate time points (*Monodelphis* days 14 and 29 and mouse day P2 and P10 postnatal), 3) late time points (mouse day P30 postnatal and *Monodelphis* day 63 and adult), and, 4) *Monodelphis* day 35 and mouse day E18 (Fig. 6c). Integration with GSE74243 recapitulated these results (Fig. 6b and d) showing overall a broadly conserved temporal signature of gene expression during mammalian lung development.

**Fig. 7:**
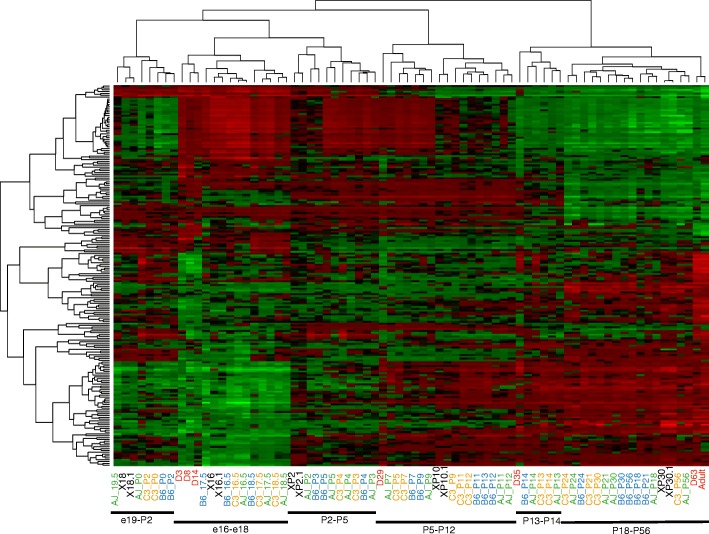
Clustering of correlated gene expression profiles during lung development in monodelphis and mice. Heatmap of 207 genes with highly correlated (correlation > 0.8) gene expression changes during lung development in *Monodelphis* (red labels) and mice GSE20954 (black labels) and GSE74243 (blue); centered and scaled log expression values. GSE74243 contains data from 3 strains of mice (green AJ, blue B6 and orange C3). Here we used the average of the 3 animal replicates to summarise expression values for each strain at each type point. The grouping of mice data is represented at the bottom

The interspecific correlation of temporal gene expression was estimated by matching the gene expression average of each species during early, intermediate and late development. Of 510 differentially expressed genes identified in both species, 207 genes had highly correlated expression profiles (Pearson product-moment correlation coefficient [cor] > 0.8, including 142 genes with cor > 0.9). This included lung specific genes (*AGER* and *PRX*) and genes with established roles in mammalian lung development*.* The gene set was associated with secreted glycoprotein and the extracellular matrix (Enrichment Score: 6, corrected *p*-value 1E-5 ~ 8.4E-7), cellular division, insulin-like binding proteins and EGF signalling, proteolysis, collagen metabolism and immune response (Enrichment Score: 3), immune system process, system development, T cell regulation (Enrichment Score: 2), cell-cell adhesion, blood circulation, cell cycle, WNT signalling, development, muscle fibres and transcription (Enrichment Score < 3). It can be seen in Fig. 7 that early *Monodelphis* day 3, 8 and 14 co-clustered with embryonic E16 to E18.5 in mice. *Monodelphis* D29 co-clustered with P3 to P13 in mice while *Monodelphis* day 35 co-clustered in a late cluster from day P13–14 postnatal in mice and included the late samples from both *Monodelphis* (day 63 and adult) and mice (up to day P56). This alignment of the major phases of lung development in mice and *Monodelphis* further supports the conserved progression of a significant proportion of the lung transcriptome during development.

### Differential temporal expression between *Monodelphis* and mice

In Fig. 7, the major specific difference was during the perinatal phase in mice with a clear separation between E19-P2 and P3-P5. The perinatal period E19.5-P2 was unusual in the mouse with an apparent temporary down-regulation of genes involved in cellular division and upregulation of genes involved in muscle formation. In contrast no such down regulation was seen in *Monodelphis* until day 35 with the activation of alveologenesis, although we cannot exclude that a similar effect occurs at an intermediate time between day 14 to 35 in *Monodelphis*.

More generally, genes with low temporal correlation between the species were associated with secreted and signalling proteins (Enrichment Score: 10, corrected *p*-value 1E-9 ~ 1E-16), immunity (adaptive and innate, Enrichment Score: 6, corrected *p*-value 1E-5 ~ 2.4E-7), muscle proteins (Enrichment Score: 3.5, corrected *p*-value 1E-5 ~ 6E-9), proteolysis (Enrichment Score: 3.5, corrected *p*-value 1.5E-2 ~ 7.8E-5), extracellular matrix (Enrichment Score: 3, p_value 6.3E-9) and weakly associated with platelet activation and positive regulation of vascular endothelial growth factor production. To retrieve genes differentially expressed between the species a second approach was employed. Genes with high loadings of the second PCA component (representing species variation) were retrieved (77 genes with absolute loadings above 0.04, including 59 genes identified as differentially expressed in *Monodelphis* with 32 genes differentially expressed in both species). Functional enrichment analysis identified two clusters. One cluster was enriched in genes associated with blood coagulation (*VTN, FGA, FGB, FGG, AMBP, APOH*, fold enrichment 28.81, *p*-value 3.72E-02) representing genes highly expressed in the early postnatal period and the functionalization of the lung in both species and therefore directly correlated with time of birth rather than development progress. This cluster also contained *ANG* (angiogenin), an important regulator of angiogenesis, and *WISP1* (WNT1-inducible signaling protein-1), a gene known to be important in lung maintenance and repair through the WNT signalling pathway [27]. *WISP1* was highly expressed postnatally from day 3 to 35 in *Monodelphis* and from P3 to P14 in the mouse. The second cluster was enriched in genes associated with muscle contraction (10 genes, fold enrichment 23.48, p-value 4.16E-09; myosin light chain 2 [*MYL2],* small muscle protein, X-linked *SMPX,* myomesin 2 *MYOM2*, myosin light chain, phosphorylatable, fast skeletal muscle *MYLPF,* myosin light chain 1 *MYL1,* myosin binding protein C, slow type *MYBPC1,* troponin T1, slow skeletal type *TNNT1*, tropomodulin 4 *TMOD4,* leiomodin 3 *LMOD3* and myosin light chain 10 *MYL10*), which are highly expressed in late stages (day 35 to adult) in *Monodelphis* and generally poorly expressed in mice. These results therefore also point to a differential regulation of blood coagulation factors and muscle fibres between the two species.

## Discussion

### Breathing with immature lungs

In eutherians such as humans, a primitive lung structure is present in highly preterm young and the mortality rate in these infants is higher, especially in very preterm infants [28, 29]. There are important determinants such as country of birth, race and socioeconomic status that affect the rate [30–32], but the underlying pathology is most often due to immature lung development in the preterm infants [33–35]. In the infants that survive, the consequence of immaturity of the lungs at birth may be significant developmental problems [30, 32, 36]. At birth, the lung of the newborn opossum is comprised of thin-walled large air sacs with few sacs occupying the whole organ. Until day 8 of post-natal development, the lungs contain few air sacs with walls of epithelial cells connected by primitive respiratory ducts. Overall, marsupial opossum neonatal lungs are immature at birth and the respiratory tree is still undergoing considerable post-natal development during the lactation period. In contrast to eutherian newborns, respiration in marsupial neonates occurs through the skin and low metabolic activity allows neonatal survival in the absence of fully functional lungs [12, 14]. Here, morphology and gene expression profiles of embryonic mice E16 and *Monodelphis* days 3 to 8 were the most similar in the pre-saccular stage.

### Signaling pathways and their significance in lung development

Lung organogenesis is primarily dependant on epithelial-mesenchymal interactions [37–39]. These interactions are mainly mediated by secretory factors released from epithelial and mesenchymal cells [40]. Transcriptional factors, growth factors, ECM proteins and MMPs mediate the interactions and participate in regulatory feedback loops [37–39]. Many of the genes contributing to pathways known to influence lung development in eutherians are also regulated in *Monodelphis,* indicating a large overlap between mechanisms of lung development in marsupials and eutherians. These pathways include Wnt [41, 42], retinoic acid [43], TGFβ [44] and NOTCH signalling pathways [45]. Although it is difficult to draw conclusions from bulk organ gene expression profiling, some candidate genes possibly contributing to these developmental pathways, angiogenesis and ECM remodelling are discussed in more details in Additional file 3.

### Conserved processes of lung development

There are limitations in the comparative analysis of gene expression during lung development in *Monodelphis* and mice. For technical reasons the study had to focus on the variation of gene expression and relied on current annotation. However a significant proportion (~ 20–30%) of RNAs identified remains anonymous. Despite these limitations and the potential difference in specific lung development dynamics, the study has shown largely conserved transcriptome dynamics during mammalian lung development allowing the filtering of some of the most important putative signalling factors. The data also indicated the major influence on bulk RNA-seq of the transition from saccular to alveolar stages rather than time from birth, as has been previously suggested from eutherian studies alone [23].

### Specific differences and their significance in lung development

Discordant expression patterns between the species were likely to be related to physiological differences between the embryonic eutherian and the partially functional lung of marsupial neonates. Differences related to blood circulation, immunity, enrichment in platelet activation and vascular proliferation probably reflect the activation of lung circulation at birth following constriction of the ductus arteriosus shortly after birth, which takes place at different stages of development in marsupial and eutherians. This is also supported by recent characterisation of the role of the lung as a major site of platelet biogenesis and a reservoir for haematopoietic progenitors [46]. Differences in the extracellular matrix are likely to denote the evolutionary flexibility of this multi-component system made in part of collagens, proteases and protease inhibitors. This is also supported by reports of differential expression of ECM components by different strains of mice and between mice and human lung in embryonic stages [26]. Similarly, enrichment in muscle contraction is compatible with more extensive differentiation of smooth muscle cells in lung tissue of marsupials compared to eutherians [13]. Specific up-regulation from the onset of alveologenesis to the mature stage only in *Monodelphis* is supported by qPCR validation of myotilin (*MYOT)* expression (*n* = 3 at each time points) and included a number of contractile protein candidate genes such as actin and myosins (e,g, *ACTA1, MYL1, MYL2, MOYM2*). Because the lung is isolated by pleural membranes, contamination of samples from skeletal muscle tissue is unlikely, especially in older animals with larger lungs. This observation raises the issue of the role of contractile fibres in the origin and evolution of the mammalian bronchoalveolar lung. Indeed, a variety of muscular lung morphological associations have been described in the multicameral lung of non-mammal tetrapods which is regarded as the precursor of the mammalian bronchoalveolar lung [47]. It has been argued that, like the appendix, lung muscle cells are a vestigial remnant without function in the lung [48]. However, recent studies have supported the role of smooth muscle cells and myofibroblasts in lung development and remodelling, including the essential role of localized smooth muscle cell differentiation for epithelial bifurcation during branching morphogenesis [49] and the role of YAP (YY1 associated protein 1) in regulating mechanical force through the phosphorylation of myosin light chains [50]. Alveolar myofibroblasts make an essential contribution to alveolar septal formation during alveologenesis [51] and 3D microscopy has recently shown how myofibroblasts deposit overlapping fishnet-like networks of actin and elastin fibres to define the walls of the developing alveoli, emphasising the crosstalk between the contractile properties of myofibroblasts and the mechanical properties of the extracellular matrix [52]. These observations clearly establish the role of muscular contraction in lung morphogenesis and support the concept that mechanical forces may have contributed to the evolution of the bronchoalveolar lung of mammals. Interestingly, the expression of skeletal myosin heavy chains was observed in rat lung myofibroblasts in vitro, and the expression control differed from that in muscle [53], suggesting that eutherian lung myofibroblasts have the capacity to be reprogramed to express a skeletal muscular protein. However, eutherian alveolar myofibroblasts typically apoptose after alveolarisation, although studies have implicated these cells in lung tissue repair and a number of serious medical conditions characterised by elastin fibre deposition. The lung contains about 40 cell types and myofibroblasts represent 10% of mature lung cells. As the normal rate of regeneration is estimated at 5% lung/week, a better understanding of the functional evolution of the mammalian lung myofibroblast could further improve our understanding of lung physiology. However, the exact production, localisation and function of these putative contractile proteins in *Monodelphis* and other species remain to be fully established.

Finally, growth and development of the marsupial neonate may, in part, be regulated by the timely delivery of maternal signalling factors supplied through milk [54–56]. Remarkably, day 60 tammar milk induced the differentiation of mouse lung mesenchyme cell cultures into invasive cells resembling smooth muscle cells or myofibroblasts [57]. Whether this differentiation results in the activation of muscular protein gene expression remains to be investigated.

## Conclusion

Marsupials provide a unique animal model to improve our understanding of lung development. Gene expression profiling of postnatal lung development in *Monodelphis* has identified markers and candidate genes with putative physiological or regulatory roles in lung development. However, the full extent of the contribution of many of these genes is still unknown and additional studies will be required to fully assess their role and improve the temporal resolution with additional time points or single cell transcriptomics. Overall the study has shown a large overlap in gene expression during lung development of the marsupial *Monodelphis* and the eutherian mouse despite differences mainly due to the timing of birth and contractile protein gene expression. The study shows how similar pathways are likely involved in the control of lung development of marsupials and eutherians and highlights the distinctive value of marsupial models towards understanding the evolution of mammalian lung development and the further identification of marsupial milk factors and their putative uterine equivalent in eutherians.

## Methods

### Lung tissue sample collection and ethics statement

The South American gray short-tailed opossum were provided by the colony established at Melbourne University. Lungs were collected from neonates on day 1, 3, 6, 8, 12, 14, 18, 24, 29, 35, 41, 51, 61, 100 of age and from an adult (Animal Ethics approval ID 1112115 from the University of Melbourne Animal Ethics committee Anatomy & Neuroscience, Pathology, Pharmacology and Physiology). The age of the neonates was determined by checking the breeding females on a daily basis after mating and removing young at appropriate ages after birth. Whole lungs were isolated by dissection and washed in PBS to remove any blood cells before further processing of the tissue.

### Tissue processing for histology

For histology, whole lungs collected from one animal at each time point (postnatal day 1, 3, 6, 12, 18, 24, 35, 41, 51, 61 and adult) were fixed in 10% neural buffered formalin for 24-48 h and left in 70% ethanol prior to further processing. All tissue samples were routinely processed using ascending ethanol series and xylene before embedment in paraffin wax. Tissue samples were sectioned on a microtome in 4–6 μm thick slices and stained with Gill’s haematoxylin & eosin stains.

### RNA sequencing

After PBS washing of whole lung dissected from one animal at each time point (day 3, 8, 14, 29, 35, 63 of development and adult), clean scalpel blades were used to cut the lung into smaller pieces before homogenisation. RNA isolation was performed using an Ambion RNA isolation kit and the quality and integrity were confirmed using the Agilent 2100 Bioanalyser RNA Nano Chip. RNA samples were sequenced using the Illumina Hiseq 2000 RNA-seq sequencing platform at BGI Co., Ltd. From 24 to 38 million Illumina paired-end read pairs were obtained in each sample. Paired-end raw RNA-seq reads were cleaned by removing reads with low quality, adaptors only reads or reads with unknown nucleotides larger than 5% of read length. Filtered RNA reads were then aligned to the *Monodelphis* genome (Monodelphis_domestica_broad05_67.gtf annotation from Ensembl) by, samtools-0.1.18, bowtie2–2.0.0-beta6, tophat-2.0.3 and Cufflinks2.1.1.1 software tools. From 20 to 31 million read pairs could be successfully mapped and, out of 33,996 referenced genes in the genome reference, 30,750 genes were identified, including 25,854 genes annotated with an official gene symbol (82%). The mapped RNA-seq data was then analysed using SeqMonk (version 0.24.1). The data was subjected to the RNA-Seq pipeline to define probes and quantify expression; the probes were defined through the probe generator, selecting mRNA features and removing exact duplicates and probes with no data, and the RNA-seq quantification pipeline was used for quantification (including mRNA features selection, log transform of normalised counts and including duplicate reads only once). Statistical filtering by intensity difference (*P* < 0.05) identified 1242 genes as significantly differentially expressed. Exploratory gene expression clustering was performed in SeqMonk, Hierarchical Clustering Explorer and R software.

### C-DNA synthesis and q-PCR

One microgram of total RNA per sample was used to synthesise cDNA. Superscript III™ Reverse Transcriptase (Invitrogen) was used to synthesise cDNA following manufacturer instructions. Real time Q-PCR was performed using SsoFast EvaGreen Supermix (Bio-Rad) and CFX96TM Real-Time PCR Detection System (Bio-Rad). The reaction mix contained diluted cDNA, 1X master mix and Forward and Reverse primers (Table 1). Sequencing results were validated by RT-PCR. A set of 10 genes was selected to represent differential expression clusters (genes chosen from profiling of Fig. 3). In addition, 6 housekeeping genes (*GAPDH, 18S, RPL19, PPIL4, GUSB* and *ACTB2*) were used as control for data normalisation. After standardization of housekeeping gene expression, 3 genes were retained for qPCR normalisation (*18S, RPL19* and *PPIL4*). At each time point, PCR assays were performed on three individual whole lung samples from different animals.

**Table 1:** List of primers for genes used in q-PCR analysis to validate RNA-seq data

Gene Title	Forward Primer	Reverse Primer
MYOT	GCCTCAGATGCAGGAACTTAT	CCAGAATATCTTTGGTGGAGGT
DSG1	AAACTACCTCGAGTGGCGTG	AACAGGCTCAAAGCCTCCTC
CA3	GCTTCACCTGGTTCACTGGA	AAGCCGGGAAAAGGCAAGAT
SPINK5	GTGACCCTGTACGTGATGCT	TGACTGGGTCACTCTCTCGT
SERPINC1	AGCAGCTCATGGAGGTGTTC	AGCTTGGCTCCATATACCGC
18S	CCCGAGAAGTTCCAGCACAT	TGATAGTGATCACGCGCTCC
RPL19	GTATGGCTTGACCCCAACGA	CTTCTCAGGCATACGGGCAT
PPIL4	ACAATGGCAGTGATCAACATGG	AAGCCAGCTGGGTCATCAAA
ARSF	ACATTCGCAGCCACCCTAAA	AGGATCTGGCCAACAAGAGC
FGA	CCCTCACAGAGAAGCAAGCA	CATCATCACAGTCTCGGGCA
HPX	CCGACGGTTTAACCCTGTGA	CATCCCTCCCTGAGTCCAGA
HTRA1	CAGTAGCGAGCCGGTGTG	TGGAAAAGGGTAGCTTGCGA
TNC	GGCTTTAGCACACACCCTCT	GGGTTTCAGCTTGGAGGTCA

### Gene clustering and functional categorisation

Statistical analysis using SeqMonk identified a total of 1242 genes had significant (*P* > 0.05) differential expression during development. These genes were subjected to clustering using Hierarchical Clustering Explorer and R. Heat maps were generated. The PANTHER™ Classification System (http://www.pantherdb.org/) and DAVID Bioinformatics Resources 6.7 (http://david.abcc.ncifcrf.gov/home.jsp) were used to perform functional categorisation and gene set enrichment analysis. Comparison with mice expression microarray was performed in R/Bioconductor. To compare lung development of the eutherian mouse and *Monodelphis*, the Gene Expression Omnibus database (GEO) was searched for a published reference dataset of gene expression during mouse lung development. Unfortunately, RNA-Seq data were not yet available for the mouse or any other eutherian mammals. However, two gene expression microarray datasets were available; 1) a study of 7 time points including 5 time points covering the time interval between the saccular and alveolar phases (GEO dataset GSE20954) [25, 26] and 2) a more recent comprehensive analysis of 3 strains of mice with 26 time points, including 20 time points in the saccular and alveolar phases (GSE74243) [25, 26]. Comparative analysis of *Monodelphis* RNA-seq data and mouse microarray datasets is compounded by the difference in technology (digital versus analogue signals with RNA-seq and microarray respectively), the limited annotation of the *Monodelphis* genome, as well as the difference in the timing of development stages between the two species. Nevertheless, 11,391 ortholog gene expression profiles common to the *Monodelphis* and mouse datasets were retrieved. Microarray data do not generally allow the direct quantitative comparison of signal intensities of different probes. Therefore, a simple normalisation step was applied independently in each dataset to each gene. Log transformed expression values were normalised by background correction and centring the samples on the mean to align the distributions followed by centring the expression of each gene in each species independently on mean expression to perform principal components analysis, clustering and estimate correlation of gene expression profiles during development.

## Additional files

Additional file 1: **Figure S1.** High resolution H&E staining of lung tissue samples collected from *Monodelphis* at different time points of postnatal development. Similarly to Fig. 1, based on morphology, the new born (A) were in the canalicular stage, lungs collected at day 8 (B) and day 14 (C) were at early saccular stage, and at saccular stage by day 29 (D) and day 35 (E), while at day 61 (F) lungs were mature with an increased alveolar number similar to an adult lung (G). Scale bar 1 mm. See also Fig. 1 for pictures at lower resolution. (PDF 21560 kb)

Additional file 2: **Figure S2.** Pie charts representing the functional categorisation of 1242 differentially expressed genes during *Monodelphis* lung development (molecular function and biological process). (A) Functional annotation based on protein class. (B) Functional annotation based on biological process. (C) Sub-classification of genes involved in development processes. (PDF 660 kb)

Additional file 3: Detailed analysis of signaling pathways and their significance in *Monodelphis* lung development. (DOCX 88 kb)

## Abbreviations

ACOXL: Acyl-CoA oxidase like
ACTA1: Actin, alpha 1, skeletal muscle
ACTA2: Actin, alpha 2, smooth muscle, aorta
ACTB2: Actin Beta 2
ACTG2: Actin, gamma 2, smooth muscle, enteric
ADA: Adenosine deaminase
ADSSL1: Adenylosuccinate synthase like 1
AGER: Advanced glycosylation end-product specific receptor
AKAP5: A-kinase anchoring protein 5
ALDH1A2: Aldehyde dehydrogenase 1 family member A2
AMBP: Alpha-1-microglobulin/bikunin precursor
ANG: Angiogenin
ANG1: Angiopoietin 1
ANG1: Transmembrane 7 superfamily member 2
ANG2: Angiopoietin 2
ANG2: VPS51, GARP complex subunit
ANGPTL3: Angiopoietin-like 3
ANGPTL4: Angiopoietin-like 4
APOH: Apolipoprotein H
ARSF: Arylsulfatase F
BGI: Beijing Genomic Institute
BMP: Bone morphogenic protein
BPIFA1: BPI fold containing family A member 1
CA3: Carbonic anhydrase III
CAPSL: Calcyphosine like
CCL20: C-C motif chemokine ligand 20
CCN1: Cyclin A2
CCN1: Cysteine rich angiogenic inducer 61
CDH11: Cadherin 11
CDHR3: Cadherin related family member 3
CDHR4: Cadherin related family member 4
cDNA: Complementary deoxyribonucleic acid
CFAP65: Cilia and flagella associated protein 65
CLDN18: Claudin 18
COL11A1: Collagen type XI alpha 1 chain
COL12A1: Collagen type XII alpha 1 chain
COL13A1: Collagen type XIII alpha 1 chain
COL16A1: Collagen type XVI alpha 1 chain
COL1A1: Collagen type I alpha 1 chain
COL3A1: Collagen type III alpha 1 chain
COL4A1: Collagen type IV alpha 1 chain
COL4A2: Collagen type IV alpha 2 chain
COL4A3: Collagen type IV alpha 3 chain
COL4A4: Collagen type IV alpha 4 chain
COL5A1: Collagen type V alpha 1 chain
COL5A3: Collagen type V alpha 3 chain
COL6A2: Collagen type VI alpha 2 chain
COL6A6: Collagen type VI alpha 6 chain
COL9A1: Collagen type IX alpha 1 chain
COL9A2: Collagen type IX alpha 2 chain
COL9A3: Collagen type IX alpha 3 chain
cor: Pearson product-moment correlation coefficient
CRABP2: Cellular retinoic acid binding protein 2
CTGF: Connective tissue growth factor
CYP2F1: Cytochrome P450 family 2 subfamily F member 1
CYR61: Cysteine rich angiogenic inducer 61
DCN: Decorin
DHRS9: Dehydrogenase/reductase 9
DLL4: Delta like canonical Notch ligand 4
DSG1: Desmoglein-1
ECM: Extracellular matrix
EGF: Epidermal growth factor
EPAS1: Endothelial PAS domain protein 1
ES: Enrichment score
FAM92B: Family with sequence similarity 92 member B
FGA: Fibrinogen alpha chain
FGB: Fibrinogen beta chain
FGG: Fibrinogen gamma chain
FZD2: Frizzled class receptor 2
GAPDH: Glyceraldehyde-3-phosphate dehydrogenase
GEO: Gene expression omnibus
GKN2: Gastrokine 2
GUSB: Glucuronidase beta
HPX: Hemopexin
HTRA1: Serine protease HTRA1
IGF: Insulin growth factor
IGF2BP1: Insulin like growth factor 2 mRNA binding protein 1
IGF2BP2: Insulin like growth factor 2 mRNA binding protein 2
IGF2BP3: Insulin like growth factor 2 mRNA binding protein 3
IGFBP1: Insulin like growth factor binding protein 1
IGFBP2: Insulin like growth factor binding protein 2
IGFBPs: Insulin like growth factor binding proteins
KRT4: Keratin 4
LAMA4: Laminin subunit alpha 4
LAMP3: Lysosomal associated membrane protein 3
LDLRAD1: Low density lipoprotein receptor class A domain containing 1
LEF1: Lymphoid enhancer binding factor 1
LMOD3: Leiomodin 3
LRRN4: Leucine rich repeat neuronal 4
MMP14: Matrix metallopeptidase 14
MMP16: Matrix metallopeptidase 16
MMP2: Matrix metallopeptidase 2
MMP8: Matrix metallopeptidase 8
MMP9: Matrix metallopeptidase 9
MMPs: Matrix metallopeptidases
MOYM2: M-protein, also known as myomesin-2
MS4A15: Membrane spanning 4-domains A15
MUC5B: Mucin 5B, oligomeric mucus/gel-forming
MYBPC1: Myosin binding protein C, slow type
MYH7: Myosin heavy chain 7
MYH7B: Myosin heavy chain 7B
MYL1: Myosin light chain 1
MYL10: Myosin light chain 10
MYL2: Myosin light chain 2
MYLPF: Myosin light chain, phosphorylatable, fast skeletal muscle
MYOM2: Myomesin 2
MYOT: Myotilin
NAPSA: Napsin A aspartic peptidase
NFAT: Nuclear factor of activated T-cells
NOTCH: Notch signaling pathway
NOTCH4: Notch 4
NOXO1: NADPH oxidase organizer 1
PAS: Per-Arnt-Sim domain
PCA: Feline leukemia virus subgroup C cellular receptor 1
PCDH18: Protocadherin 18
PCR: Polymerase chain reaction
PDGF: Platelet-derived growth factor
PPIL4: Peptidylprolyl isomerase like 4
PRX: Periaxin
PRX: Peroxiredoxin 6
qPCR: Quantitative PCR
RA: Retinoic acid
RETNLB: Resistin like beta
RNA: Ribonucleic acid
RNAseq: Ribonucleic acid sequencing
RPL19: Ribosomal protein L19
RTKN2: Rhotekin 2
RTPCR: Reverse transcriptase polymerase chain reaction
SCGB3A1: Secretoglobin family 3A member 1
SCGB3A2: Secretoglobin family 3A member 2
SEC14L3: SEC14 like lipid binding 3
SERPINC1: Serpin family C member 1
SFRP: Secreted frizzled related protein
SFRP1: Secreted frizzled related protein 1
SFRP2: Secreted frizzled related protein 2
SFTPB: Surfactant protein B
SFTPC: Surfactant protein C
SLC6A14: Solute carrier family 6 member 14
SMPX: Small muscle protein, X-linked
SNTN: Sentan, cilia apical structure protein
SPARC: Secreted protein acidic and cysteine rich
SPINK: Serine protease inhibitor Kazal-type
TCF7: Transcription factor 7
TGFB2: Transforming growth factor beta 2
TMOD4: Tropomodulin 4
TNC: Tenascin C
TNFAIP2: TNF alpha induced protein 2
TNNT1: Troponin T1, slow skeletal type
VEGF: Vascular endothelial growth factor A
VTN: Vitronectin
WIF1: WNT inhibitory factor 1
WISP1: WNT1 inducible signaling pathway protein 1
WNT: Wnt signaling pathways
YAP: YY1 associated protein 1
