# Supplementary figures and images for: Gene expression profiling of postnatal lung development in the marsupial gray short-tailed opossum (Monodelphis domestica) highlights conserved developmental pathways and specific characteristics during lung organogenesis
Source: BMC Genomics. 2018 Oct 5;19:732. doi: 10.1186/s12864-018-5102-2 (PMC6173930; doi:10.1186/s12864-018-5102-2)

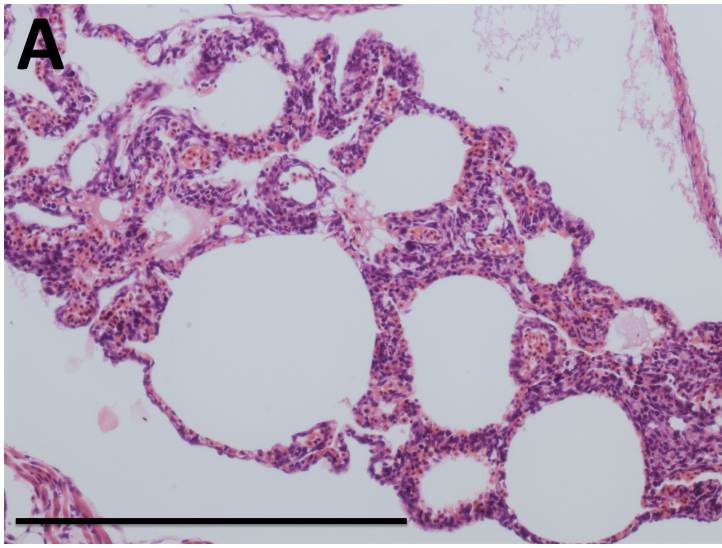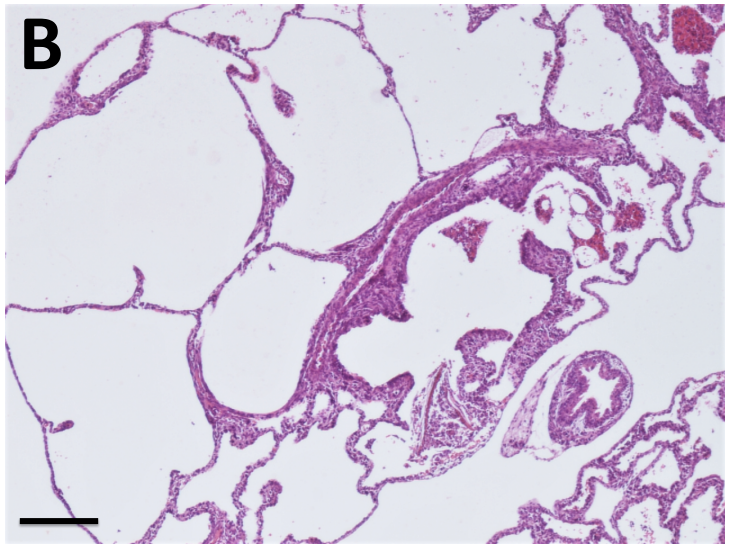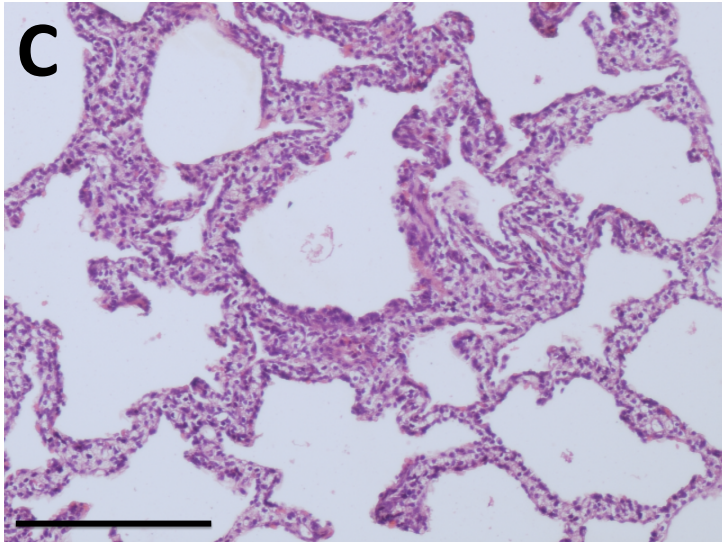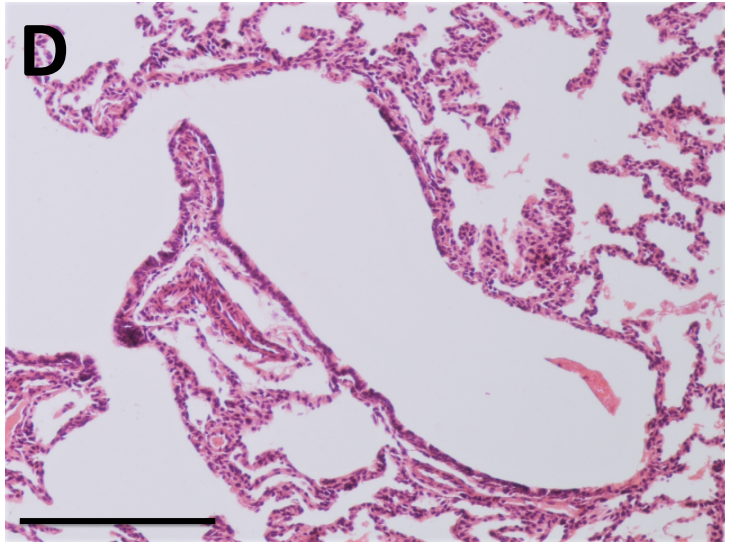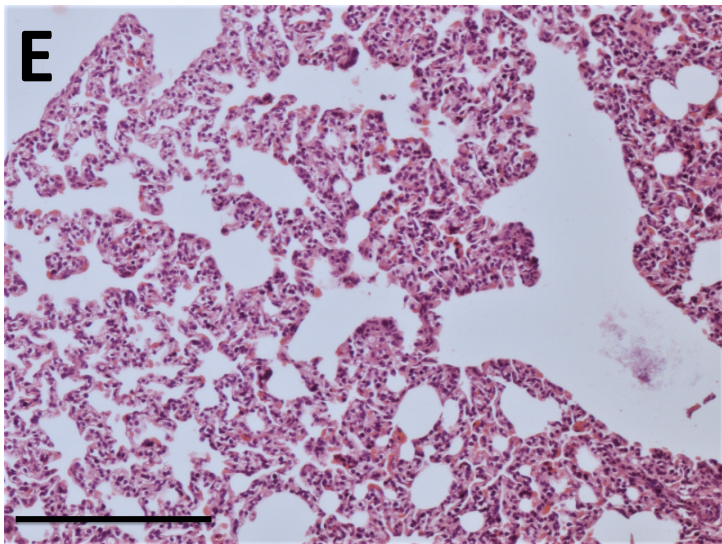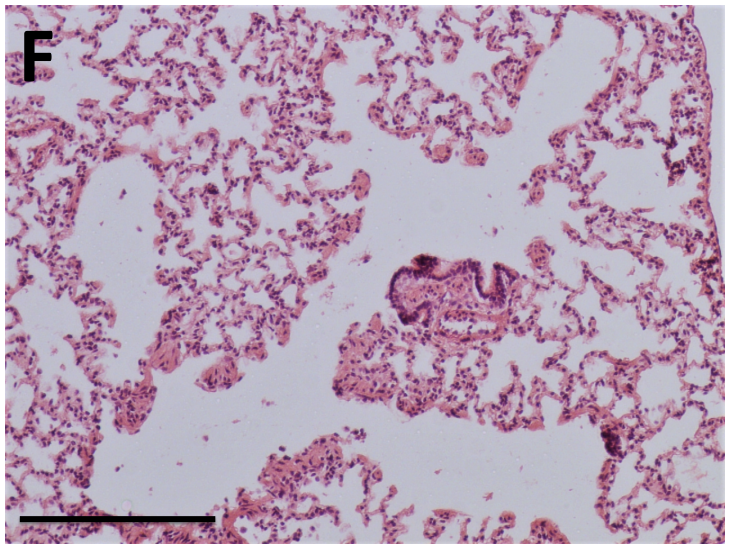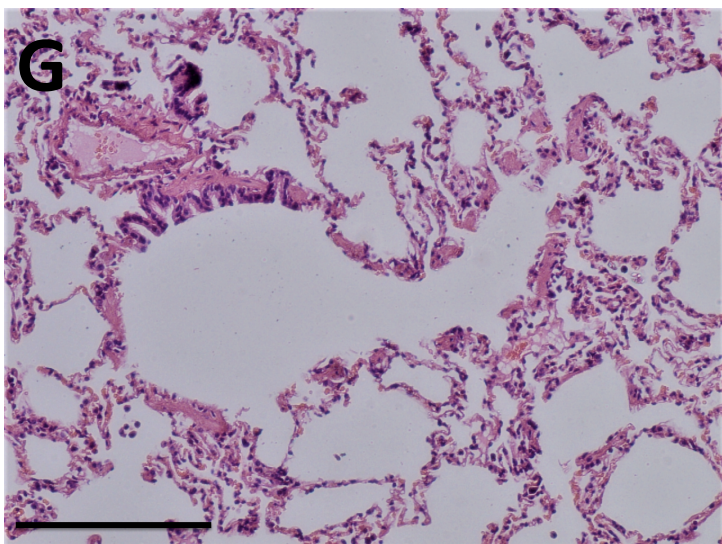

Supplement: Supplementary file 1 — Figure S1. High resolution H&E staining of lung tissue samples collected from Monodelphis at different time points of postnatal development. Similarly to Fig. 1, based on morphology, the new born (A) were in the canalicular stage, lungs collected at day 8 (B) and day 14 (C) were at early saccular stage, and at saccular stage by day 29 (D) and day 35 (E), while at day 61 (F) lungs were mature with an increased alveolar number similar to an adult lung (G). Scale bar 1 mm. See also Fig. 1 for pictures at lower resolution. (PDF 21560 kb) [file 12864_2018_5102_MOESM1_ESM.pdf]
