# Supplementary material for: Gene expression profiling of postnatal lung development in the marsupial gray short-tailed opossum (Monodelphis domestica) highlights conserved developmental pathways and specific characteristics during lung organogenesis
Source: BMC Genomics. 2018 Oct 5;19:732. doi: 10.1186/s12864-018-5102-2 (PMC6173930; doi:10.1186/s12864-018-5102-2)
Supplement: Supplementary file 2 — Figure S2. Pie charts representing the functional categorisation of 1242 differentially expressed genes during Monodelphis lung development (molecular function and biological process). (A) Functional annotation based on protein class. (B) Functional annotation based on biological process. (C) Sub-classification of genes involved in development processes. (PDF 660 kb) [file 12864_2018_5102_MOESM2_ESM.pdf]

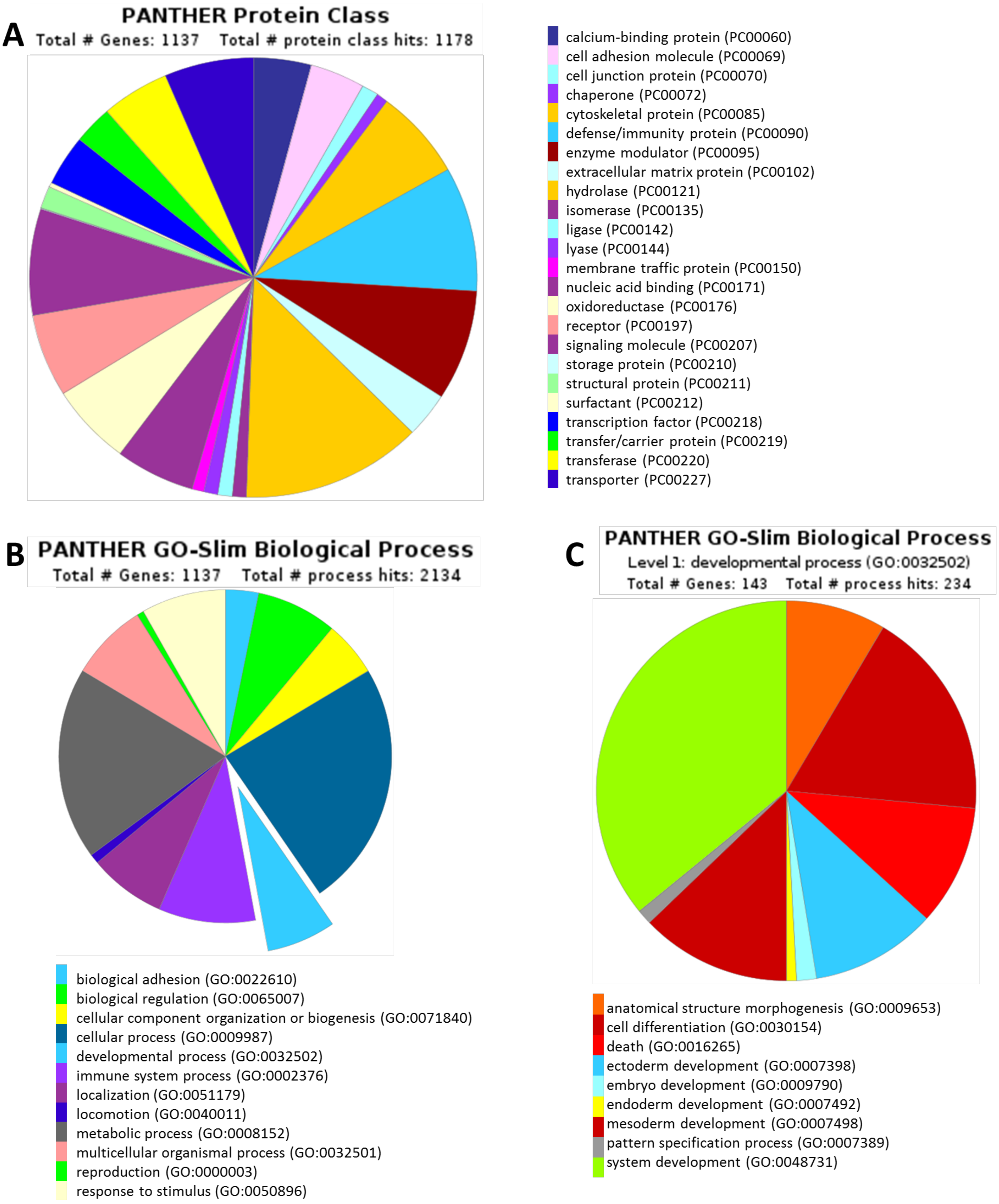

A
PANTHER Protein Class
Total # Genes: 1137 Total # protein class hits: 1178
calcium-binding protein (PC00060)
cell adhesion molecule (PC00069)
cell junction protein (PC00070)
chaperone (PC00072)
cytoskeletal protein (PC00085)
defense/immunity protein (PC00090)
enzyme modulator (PC00095)
extracellular matrix protein (PC00102)
hydrolase (PC00121)
isomerase (PC00135)
ligase (PC00142)
lyase (PC00144)
membrane traffic protein (PC00150)
nucleic acid binding (PC00171)
oxidoreductase (PC00176)
receptor (PC00197)
signaling molecule (PC00207)
storage protein (PC00210)
structural protein (PC00211)
surfactant (PC00212)
transcription factor (PC00218)
transfer/carrier protein (PC00219)
transferase (PC00220)
transporter (PC00227)
B
PANTHER GO-Slim Biological Process
Total # Genes: 1137 Total # process hits: 2134
biological adhesion (GO:0022610)
biological regulation (GO:0065007)
cellular component organization or biogenesis (GO:0071840)
cellular process (GO:0009987)
developmental process (GO:0032502)
immune system process (GO:0002376)
localization (GO:0051179)
locomotion (GO:0040011)
metabolic process (GO:0008152)
multicellular organismal process (GO:0032501)
reproduction (GO:0000003)
response to stimulus (GO:0050896)
C
PANTHER GO-Slim Biological Process
Level 1: developmental process (GO:0032502)
Total # Genes: 143 Total # process hits: 234
anatomical structure morphogenesis (GO:0009653)
cell differentiation (GO:0030154)
death (GO:0016265)
ectoderm development (GO:0007398)
embryo development (GO:0009790)
endoderm development (GO:0007492)
mesoderm development (GO:0007498)
pattern specification process (GO:0007389)
system development (GO:0048731)
