# Supplementary material for: Gene expression profiling of postnatal lung development in the marsupial gray short-tailed opossum (Monodelphis domestica) highlights conserved developmental pathways and specific characteristics during lung organogenesis
Source: BMC Genomics. 2018 Oct 5;19:732. doi: 10.1186/s12864-018-5102-2 (PMC6173930; doi:10.1186/s12864-018-5102-2)
Supplement: Supplementary file 3 — Detailed analysis of signaling pathways and their significance in Monodelphis lung development. (DOCX 88 kb) [file 12864_2018_5102_MOESM3_ESM.docx]

**Additional file 3**

**Detailed analysis of signaling pathways and their significance in *Monodelphis* lung development.**

Several developmental pathways are known to regulate foetal lung development in eutherians. These pathways include Wnt [[1](#_ENREF_1), [2](#_ENREF_2)], retinoic acid [[3](#_ENREF_3)] , TGFβ [[4](#_ENREF_4)] and NOTCH signalling pathways [[5](#_ENREF_5)]. It is important to realise that there are limitations in the interpretation of gene expression data from bulk organ sequencing on a large time scale with respect to developmental pathways. However, here we briefly review the differential expression of some of the genes that may contribute to these developmental pathways as well as angiogenesis and the remodelling of the extracellular matrix in both the marsupial and the eutherian models, indicating the overlap and pointing to subtle differences between mechanisms of postnatal lung development in the marsupial and foetal lung development in eutherians.

**WNT signalling**

WNT is a secreted glycoprotein controlling cell-cell communication through a wide range of functions during embryonic development including regulating cell fate and simultaneously networking with other pathways through TGFβ, NOTCH and BMP signalling [[6-8](#_ENREF_6)]. *Wnt* gene knockout studies in embryonic mice suggested WNT signalling pathways are crucial in regulating lung cell fate [[9](#_ENREF_9)]. Nuclear Factor of Activated T cells (NFAT) and WNT interactions regulate cell proliferation and differentiation as demonstrated by studies of neural progenitor cells regulation [[10](#_ENREF_10)]. WNT protein family members interact with the trans-membrane frizzled receptor to activate intracellular signal transduction and cellular response in target cells [[2](#_ENREF_2)]. The WNT signalling pathways is mediated by binding of WNT ligand to frizzled receptors [[11-13](#_ENREF_11)]. Secreted frizzled-related proteins (SFRP) are also involved in WNT signalling [[14](#_ENREF_14)].

In *Monodelphis,* differentially expressed WNT signalling members included Cadherin (*CDH11* and *PCDH18*), T-cell (*TCF7* and *LEF1*) and frizzled receptor (*FZD2*) or frizzled related proteins (*SFRP1* and *SFRP2*). Elevated expression of *CDH11* and *PCDH18* was observed during the early postnatal period (day 3 to 14) and gradually decreased during the course of lung development. *FZD2*, *SFRP1* and *SFRP2* were temporally highly expressed during the early postnatal period (day 3, 8 and 14), signifying the likely contribution of WNT/frizzled signalling to early lung development. Not surprisingly, the comparison with mice indicated that several of these genes exhibited highly correlated expression profiles (*CDH11, PCDH18, TCF7, FZD2, SFRP2*) while *SFRP1* and *LEF1* expression profiles were not correlated between the two species.

**RA signalling**

RA is known to signal embryonic development and tissue repair [[15](#_ENREF_15)]. Studies have shown RA is a key regulator of lung development and its activity was observed at different stages of eutherian foetal development including early bud formation, branching morphogenesis and lung maturation during alveolarization [[3](#_ENREF_3), [16](#_ENREF_16)]. RA also regulates alveolarization and alveolar repair by controlling elastin expression by lung fibroblast [[17](#_ENREF_17)]. Differentially expressed genes involved in RA signalling included *DHRS9 (*dehydrogenase/reductase 9)*, CRABP2 (*cellular retinoic acid binding protein 2)*, ALDH1A2* (aldehyde dehydrogenase 1 family member A2) and *TNFAIP2 (*TNF alpha induced protein 2). DHRS9 is involved in RA biosynthesis [[18](#_ENREF_18), [19](#_ENREF_19)]. CRABP2 is an additional intracellular lipid binding protein specifically binding RA [[20](#_ENREF_20)]. The expression of *CRABP2* and *DHRS9* specifically at day 63 and adult in *Monodelphis* suggests their involvement during alveogenesis and lung maturation in marsupials as previously known from eutherian studies [[3](#_ENREF_3), [21](#_ENREF_21)]. However in mice, *CRABP2, ALDH1A2* and *TNFAIP2* were apparently expressed at relatively constant levels during development.

**NOTCH signalling**

NOTCH4 signalling plays a crucial role in eutherian lung development by regulating events such as branching morphogenesis, alveolarization and the regulation of proximal and distal cell fate [[5](#_ENREF_5)]. Similarly, the temporal upregulation of *NOTCH4* at day 29 and 35 together with the morphological changes observed in *Monodelphis* support its role in regulating branching and alveolarization. Expression of *NOTCH4* was highly correlated between *Monodelphis* and mice.

**TGFβ2**

TGFβ2 is involved in diverse functions during embryonic development [[4](#_ENREF_4), [22](#_ENREF_22)] and its expression in the marsupial lung persisted until day 35, after which it reduced at day 63 and in the adult, suggesting that temporal expression of *TGFβ2* may also participate in lung organogenesis in *Monodelphis*. *TGFB2* expression was also highly correlated between *Monodelphis* and mice.

**IGF binding proteins (IGFBPs)**

IGFBPs are known to be involved in lung development [[23](#_ENREF_23), [24](#_ENREF_24)]. *IGFBP1, IGFBP2, IGF2BP1* and *IGF2BP3* displayed higher temporal expression during early postnatal day 3, 8 and day 14 in *Monodelphis* lungs. *IGFBP2* expression was previously noted both in rat and mice lung development studies [[25](#_ENREF_25), [26](#_ENREF_26)]. The expression of *IGF2BP2* and *IFG2BP3* were highly correlated between *Monodelphis* and mice. However *IGFBP1* and *IGFBP2* had dissimilar expression profiles in the two species.

**Extracellular matrix proteins (ECM) and Matrix metalloproteinases (MMPs)**

The ECM provides a complex signalling network between epithelia and mesenchyme [[27](#_ENREF_27), [28](#_ENREF_28)]. Collagens and laminins are highly expressed in lung and are involved in regulating lung morphology [[29](#_ENREF_29)]. Laminins and collagens were temporally expressed in Monodelphis. For example laminin A4 (*LAMA4*) had peak expression at day 14 and 29, concomitant with morphological changes including branching morphogenesis and increase of lung parenchyma. Collagens are also involved in branching morphogenesis. Collagen Type-I, Type-III, Type-IV and Type-V have all been shown to be regulated during foetal lung development in eutherians [[30-32](#_ENREF_30)], but the role of specific collagen isoforms is still unclear. In Monodelphis, differential expression of various collagen isoforms was also observed including *COL11A1, COL12A1, COL13A1, COL16A1, COL1A1, COL3A1, COL4A1, COL4A2, COL4A3, COL4A4, COL5A1, COL5A3, COL6A2, COL6A6, COL9A1, COL9A2* and *COL9A3*. *LAMA4* and a subset of collagens (*COL13A1, COL5A1, COL1A1, COL12A1, COL11A1, COL3A1*) were also present in the list of genes with highly correlated expression profiles in mice and cell-matrix adhesion was the second most significantly enriched biological process (9.76 fold enrichment, p=5 10^-4^) in this high cross species correlation gene set. However, a subset of ECM proteins was also differentially expressed between species.

The MMPs are also involved in lung organogenesis and mouse studies have shown MMP2 and MMP14 are involved in branching morphogenesis and alveologenesis [[33](#_ENREF_33)]. Similarly, in the present study MMP2 showed high expression levels in neonate lung compared to adult lung and temporal regulations of MMP8, MMP9 and MMP16 were also observed. In the mice microarray data MMPs were expressed at relatively constant levels excepted for MMP2 and MMP14, which displayed a highly correlated expression profile with *Monodelphis* despite MMP14 expression not being detected above the significant differential expression threshold in the analysis of *Monodelphis* data.

**Angiogenesis**

Lung vascularisation is essential for gas exchange between air and blood. The lung accommodates a complex network of blood vessels, which is initiated during the early stage of development [[34](#_ENREF_34)]. In general, the majority of vasculogenesis occurs during foetal development in eutherians [[35](#_ENREF_35), [36](#_ENREF_36)]. However, in marsupials significant development occurs after birth, including angiogenesis. Angiogenesis is also regulated by matrix and matrix related proteolitic enzymes providing structural support to extend the blood vessel network. Together the ECM and MMPs provide a favourable microenvironment to promote angiogenesis as the extending blood vessels interact with the surrounding extracellular matrix. In the current study differential expression of *COL1A1, COL3A1, COL4A1* and *LAMA4* genes in *Monodelphis* is consistent with previous studies [[37](#_ENREF_37), [38](#_ENREF_38)]. Matrix regulating proteases mediate the migration of endothelial cells by breaking down the ECM, simultaneously activating growth factors such as VEGF [[39](#_ENREF_39), [40](#_ENREF_40)]. Differential expression of MMPs in *Monodelphis* included *MMP-2, MMP-9* and *MMP-16,* which are known to contribute to angiogenesis [[39](#_ENREF_39), [41](#_ENREF_41), [42](#_ENREF_42)]. Angiogenesis is also widely regulated by transcriptional regulators and growth factors [[43](#_ENREF_43), [44](#_ENREF_44)]. A number of genes potentially related to angiogenesis have been identified in the current study including *ANG, ANGPTL3, SFRP1, SFRP2, TGFβ2, DLL4, LEF1, NOTCH4, EPAS1, CYR61 (also known as CCN1)* and *CTGF*. Angiopoietins (ANG1 and ANG2) are known to regulate vascular maturation [[45](#_ENREF_45), [46](#_ENREF_46)]. Angiopoietin-like 3 (ANGPTL3) is a member of the angiopoietin-like family involved in angiogenesis [[47](#_ENREF_47)]. The Secreted frizzled-related proteins (SFRP1 and SFRP2) also promote angiogenesis [[48](#_ENREF_48), [49](#_ENREF_49)]. From the expression data *ANG, ANGPTL3, SFRP1 SFRP2* and *TGFβ2* showed increased expression during early postnatal time points day 3 to 14, and the temporal expression of these genes in *Monodelphis* supports their participation in the stimulation of angiogenesis. In *Monodelphis DLL4*, *LEF1* and *NOTCH4* were specifically expressed at day 29 and 35. Recent studies have shown that delta-like-4 (DLL4) interacts with NOTCH to regulate angiogenesis [[50](#_ENREF_50)]. Lymphoid enhancer-binding factor 1 (LEF1) mediates the Wnt/β-catenin dependent pathway [[51](#_ENREF_51)] and promotes endothelial cell invasion [[52](#_ENREF_52)]. The NOTCH proteins and NOTCH ligands are involved in regulating the initial phase of angiogenesis and factors like TGFβ are involved in vascular maturation and remodelling [[53](#_ENREF_53)]. NOTCH4 is a transcription factor specifically expressed by endothelial cells and is a key regulator of angiogenesis [[54](#_ENREF_54)]. The differential temporal expression of these genes in *Monodelphis* lung may similarly regulate angiogenesis during lung development and the expression profiles of the majority of these genes (*NOTCH4, TGFB2, SFRP2, ANGPTL3, ANGPTL4, EPAS1, CYR61*) were highly correlated between *Monodelphis* and mice excepted for *DLL4* and *LEF1*. In addition blood circulation was also found to be an enriched biological process (5.68 fold enrichement p=8 10^-3^) in the list of highly correlated genes between *Monodelphis* and mice.

Overall, the study has largely confirmed the conserved temporal expression regulation and putative contribution of many of these important pathway mediators in postnatal lung development of *Monodelphis* while pointing to few specific differences.

1. Pongracz J, Stockley R: **Wnt signalling in lung development and diseases**. *Respiratory Research* 2006, **7**(1):15.

2. Wodarz A, Nusse R: **MECHANISMS OF WNT SIGNALING IN DEVELOPMENT**. *Annual Review of Cell and Developmental Biology* 1998, **14**(1):59-88.

3. Chytil F: **Retinoids in lung development**. *FASEB J* 1996, **10**(9):986-992.

4. Gu L, Zhu Y-j, Yang X, Guo Z-j, Xu W-b, Tian X-l: **Effect of TGF-[beta]/Smad signaling pathway on lung myofibroblast differentiation**. *Acta Pharmacol Sin* 2007, **28**(3):382-391.

5. Xu KL, Moghal N, Egan SE: **Notch Signaling in Lung Development and Disease**. *Adv Exp Med Biol* 2012, **727**:89-98.

6. Kestler HA, Kühl M: **From individual Wnt pathways towards a Wnt signalling network**. *Philosophical Transactions of the Royal Society B: Biological Sciences* 2008, **363**(1495):1333-1347.

7. Katoh M, Katoh M: **WNT Signaling Pathway and Stem Cell Signaling Network**. *Clinical Cancer Research* 2007, **13**(14):4042-4045.

8. Wang YP, Li YP, Paulson C, Shao JZ, Zhang XL, Wu MR, Chen W: **Wnt and the Wnt signaling pathway in bone development and disease**. *Front Biosci-Landmrk* 2014, **19**:379-407.

9. De Langhe SP, Reynolds SD: **Wnt signaling in lung organogenesis**. *Organogenesis* 2008, **4**(2):100-108.

10. Huang T, Xie Z, Wang J, Li M, Jing N, Li L: **Nuclear Factor of Activated T Cells (NFAT) Proteins Repress Canonical Wnt Signaling via Its Interaction with Dishevelled (Dvl) Protein and Participate in Regulating Neural Progenitor Cell Proliferation and Differentiation**. *Journal of Biological Chemistry* 2011, **286**(43):37399-37405.

11. Schulte G, Bryja V: **The Frizzled family of unconventional G-protein-coupled receptors**. *Trends in Pharmacological Sciences* 2007, **28**(10):518-525.

12. van Amerongen R, Nusse R: **Towards an integrated view of Wnt signaling in development**. *Development* 2009, **136**(19):3205-3214.

13. Logan CY, Nusse R: **The Wnt signaling pathway in development and disease**. *Annual Review of Cell and Developmental Biology* 2004, **20**:781-810.

14. Esteve P, Sandonis A, Ibanez C, Shimono A, Guerrero I, Bovolenta P: **Secreted frizzled-related proteins are required for Wnt/beta-catenin signalling activation in the vertebrate optic cup**. *Development* 2011, **138**(19):4179-4184.

15. Pittlik S, Begemann G: **New sources of retinoic acid synthesis revealed by live imaging of an Aldh1a2-GFP reporter fusion protein throughout zebrafish development**. *Developmental Dynamics* 2012, **241**(7):1205-1216.

16. Malpel S, Mendelsohn C, Cardoso WV: **Regulation of retinoic acid signaling during lung morphogenesis**. *Development* 2000, **127**(14):3057-3067.

17. Plantier L, Rochette-Egly C, Goven D, Boutten A, Bonay M, Leseche G, Fournier M, Crestani B, Boczkowski J: **Dysregulation of elastin expression by fibroblasts in pulmonary emphysema: role of cellular retinoic acid binding protein 2**. *Thorax* 2008, **63**(11):1012-1017.

18. Wang C: **Biosynthesis of all-trans-Retinoic Acid and Regulation of Retinoids Homeostasis in Primary Hippocampal Astrocytes and Neurons**. In*.*; 2010.

19. Everts HB, Sundberg JP, King LE, Ong DE: **Immunolocalization of enzymes, binding proteins, and receptors sufficient for retinoic acid synthesis and signaling during the hair cycle**. *J Invest Dermatol* 2007, **127**(7):1593-1604.

20. Moise AR, Noy N, Palczewski K, Blaner WS: **Delivery of retinoid-based therapies to target tissues**. *Biochemistry-Us* 2007, **46**(15):4449-4458.

21. Hind M, Maden M: **Retinoic acid induces alveolar regeneration in the adult mouse lung**. *Eur Respir J* 2004, **23**(1):20-27.

22. Wu MY, Hill CS: **TGF-β Superfamily Signaling in Embryonic Development and Homeostasis**. *Developmental Cell* 2009, **16**(3):329-343.

23. Shimasaki S, Ling N: **Identification and molecular characterization of insulin-like growth factor binding proteins (IGFBP-1, -2, -3, -4, -5 and -6)**. *Progress in growth factor research* 1991, **3**(4):243-266.

24. Maitre B, Clement A, Williams MC, Brody JS: **Expression of insulin-like growth factor receptors 1 and 2 in the developing lung and their relation to epithelial cell differentiation**. *American journal of respiratory cell and molecular biology* 1995, **13**(3):262-270.

25. Schuller AGP, van Neck JW, Beukenholdt RW, Zwarthoff EC, Drop SLS: **IGF, type I IGF receptor and IGF-binding protein mRNA expression in the developing mouse lung**. *Journal of Molecular Endocrinology* 1995, **14**(3):349-355.

26. Wallen L, Myint W, Nygard K, Shimasaki S, Clemmons D, Han V: **Cellular distribution of insulin-like growth factor binding protein mRNAs and peptides during rat lung development**. *Journal of Endocrinology* 1997, **155**(2):313-327.

27. Shannon JM, Hyatt BA: **Epithelial-mesenchymal interactions in the developing lung**. *Annu Rev Physiol* 2004, **66**:625-645.

28. Minoo P, King RJ: **Epithelial-mesenchymal interactions in lung development**. *Annu Rev Physiol* 1994, **56**:13-45.

29. Schuger L, Varani J, Killen PD, Skubitz APN, Gilbride K: **Laminin Expression in the Mouse Lung Increases with Development and Stimulates Spontaneous Organotypic Rearrangement of Mixed Lung-Cells**. *Developmental Dynamics* 1992, **195**(1):43-54.

30. Heine UI, Munoz EF, Flanders KC, Roberts AB, Sporn MB: **Colocalization of TGF-beta 1 and collagen I and III, fibronectin and glycosaminoglycans during lung branching morphogenesis**. *Development* 1990, **109**(1):29-36.

31. Spooner BS, Faubion JM: **Collagen involvement in branching morphogenesis of embryonic lung and salivary gland**. *Dev Biol* 1980, **77**(1):84-102.

32. Chen J-M, Little CD: **Cellular events associated with lung branching morphogenesis including the deposition of collagen type IV**. *Dev Biol* 1987, **120**(2):311-321.

33. Kheradmand F, Rishi K, Werb Z: **Signaling through the EGF receptor controls lung morphogenesis in part by regulating MT1-MMP-mediated activation of gelatinase A/MMP2**. *J Cell Sci* 2002, **115**(4):839-848.

34. Warburton D, Schwarz M, Tefft D, Flores-Delgado G, Anderson KD, Cardoso WV: **The molecular basis of lung morphogenesis**. *Mechanisms of Development* 2000, **92**(1):55-81.

35. Whitsett JA, Wert SE: **Chapter 1 - Molecular Determinants of Lung Morphogenesis**. In: *Kendig's Disorders of the Respiratory Tract in Children (Seventh Edition).* Edited by Victor C, Md, Frcpc, Thomas FB, Robert WW, Bsc, Frcp, Andrew Bush MBMMFF. Philadelphia: W.B. Saunders; 2006: 1-16.

36. Burri PH: **Fetal and postnatal development of the lung**. *Annu Rev Physiol* 1984, **46**:617-628.

37. Montesano R, Orci L, Vassalli P: **In vitro rapid organization of endothelial cells into capillary-like networks is promoted by collagen matrices**. *The Journal of Cell Biology* 1983, **97**(5):1648-1652.

38. Haas TL, Davis SJ, Madri JA: **Three-dimensional Type I Collagen Lattices Induce Coordinate Expression of Matrix Metalloproteinases MT1-MMP and MMP-2 in Microvascular Endothelial Cells**. *Journal of Biological Chemistry* 1998, **273**(6):3604-3610.

39. Stetler-Stevenson WG: **Matrix metalloproteinases in angiogenesis: a moving target for therapeutic intervention**. *The Journal of Clinical Investigation* 1999, **103**(9):1237-1241.

40. Stupack DG, Cheresh DA: **ECM Remodeling Regulates Angiogenesis: Endothelial Integrins Look for New Ligands**, vol. 2002; 2002.

41. Cornelius LA, Nehring LC, Harding E, Bolanowski M, Welgus HG, Kobayashi DK, Pierce RA, Shapiro SD: **Matrix metalloproteinases generate angiostatin: effects on neovascularization**. *Journal of immunology* 1998, **161**(12):6845-6852.

42. Dong Z, Kumar R, Yang X, Fidler IJ: **Macrophage-Derived Metalloelastase Is Responsible for the Generation of Angiostatin in Lewis Lung Carcinoma**. *Cell* 1997, **88**(6):801-810.

43. Hoeben A, Landuyt B, Highley MS, Wildiers H, Van Oosterom AT, De Bruijn EA: **Vascular Endothelial Growth Factor and Angiogenesis**. *Pharmacological Reviews* 2004, **56**(4):549-580.

44. Reinhart-King CA: **Mechanical and chemical signaling in angiogenesis**. In: *Studies in mechanobiology, tissue engineering and biomaterials,.* Berlin ; New York: Springer; 2013: 1 online resource.

45. Yu Q, Stamenkovic I: **Angiopoietin-2 Is Implicated in the Regulation of Tumor Angiogenesis**. *The American Journal of Pathology* 2001, **158**(2):563-570.

46. Jain RK: **Molecular regulation of vessel maturation**. *Nat Med* 2003, **9**(6):685-693.

47. Song M, Cho S: **Angiopoietin-like 3 (ANGPTL3) Stimulates Human Umbilical Vein Endothelial Cell Tube Formation and Vascular Endothelial Growth Factor Production**. *B Korean Chem Soc* 2009, **30**(3):707-709.

48. Dufourcq P, Descamps B, Tojais NF, Leroux L, Oses P, Daret D, Moreau C, Lamazière J-MD, Couffinhal T, Duplàa C: **Secreted Frizzled-Related Protein-1 Enhances Mesenchymal Stem Cell Function in Angiogenesis and Contributes to Neovessel Maturation**. *STEM CELLS* 2008, **26**(11):2991-3001.

49. Courtwright A, Siamakpour-Reihani S, Arbiser JL, Banet N, Hilliard E, Fried L, Livasy C, Ketelsen D, Nepal DB, Perou CM *et al*: **Secreted Frizzle-Related Protein 2 Stimulates Angiogenesis via a Calcineurin/NFAT Signaling Pathway**. *Cancer Research* 2009, **69**(11):4621-4628.

50. Kuhnert F, Kirshner J, Thurston G: **Dll4-Notch signaling as a therapeutic target in tumor angiogenesis**. *Vascular Cell* 2011, **3**(1):20.

51. Hanai J-i, Dhanabal M, Karumanchi SA, Albanese C, Waterman M, Chan B, Ramchandran R, Pestell R, Sukhatme VP: **Endostatin Causes G1 Arrest of Endothelial Cells through Inhibition of Cyclin D1**. *Journal of Biological Chemistry* 2002, **277**(19):16464-16469.

52. Planutiene M, Planutis K, Holcombe R: **Lymphoid enhancer-binding factor 1, a representative of vertebrate-specific Lef1/Tcf1 sub-family, is a Wnt-beta-catenin pathway target gene in human endothelial cells which regulates matrix metalloproteinase-2 expression and promotes endothelial cell invasion**. *Vascular Cell* 2011, **3**(1):28.

53. Felcht M, Luck R, Schering A, Seidel P, Srivastava K, Hu J, Bartol A, Kienast Y, Vettel C, Loos EK *et al*: **Angiopoietin-2 differentially regulates angiogenesis through TIE2 and integrin signaling**. *The Journal of Clinical Investigation* 2012, **122**(6):1991-2005.

54. Wu J, Iwata F, Grass JA, Osborne CS, Elnitski L, Fraser P, Ohneda O, Yamamoto M, Bresnick EH: **Molecular determinants of NOTCH4 transcription in vascular endothelium**. *Mol Cell Biol* 2005, **25**(4):1458-1474.
